# Healthcare and Economic Impact of Lung Perfusion Scintigraphy in Patients Affected by Acute Pulmonary Embolism

**DOI:** 10.3390/healthcare9060716

**Published:** 2021-06-10

**Authors:** Giuseppe Rubini, Cristina Ferrari, Paolo Mammucci, Antonio Rosario Pisani, Pierpaolo Mincarone, Carlo Giacomo Leo

**Affiliations:** 1 Section of Nuclear Medicine, Interdisciplinary Department of Medicine, University Aldo Moro of Bari, 70124 Bari, Italy; giuseppe.rubini@uniba.it (G.R.); paolo.mammucci@outlook.com (P.M.); apisani71@libero.it (A.R.P.); 2 Research Unit of Brindisi, Institute for Research on Population and Social Policies, National Research Council, 72100 Brindisi, Italy; pierpaolo.mincarone@irpps.cnr.it; 3 Institute of Clinical Physiology, National Research Council, Branch of Lecce, c/o Campus Ecotekne via Provinciale Lecce-Monteroni, 73100 Lecce, Italy; leo@ifc.cnr.it

**Keywords:** acute pulmonary embolism, lung perfusion scintigraphy, computed tomography pulmonary angiography, healthcare costs, economic model

## Abstract

Acute pulmonary embolism (APE) is a cardiovascular emergency, representing the main cause of mortality, morbidity, and hospitalisation in Europe. We aim to evaluate the economic and healthcare impact of lung perfusion scintigraphy (LPS) used in patients with suspected APE, in the event of non-conclusive or contraindicated computed tomography pulmonary angiography (CTPA). We considered two alternative healthcare processes for APE diagnosis, with and without LPS. We performed a cost analysis with the aim of evaluating the average direct healthcare costs for diagnosis, risk assessment, and treatment of APE. We used data from a monocentric trial. Our economic model showed that the strategy with LPS was preferable in terms of costs. The average per-patient costs for the diagnosis and treatment of the acute phase of PE in low-risk patients with a non-conclusive or not-executable CTPA, with and without LPS, are EUR 2145.25 and EUR 4912.45, respectively. LPS is a simple, quick, and economic examination, useful in this setting of patients not only for an early diagnosis but also to exclude APE, demonstrating an advantage in terms of healthcare resources. To the best of our knowledge, this study is the first to analyse the economic and healthcare impact of the use of LPS in the diagnostic pathway of suspected APE.

## 1. Introduction

Acute pulmonary embolism (APE) is a cardiovascular emergency defined as the sudden obstruction of a pulmonary artery, mainly caused by a thrombus-derived embolus developing in the venous system of the lower limbs in the presence of deep vein thrombosis (DVT) [1]. It represents the third most common cardiovascular disease (after myocardial infarction and stroke) and the main cause of mortality, morbidity, and hospitalisation in Europe, as well as maternal mortality [2]. In Western countries, the yearly incidence of APE is 50 events per 100,000 inhabitants, whereas in Italy, it is higher in women than in men, with 55.4 and 40.6 events per year per 100,000 inhabitants, respectively (*p* < 0.001) [3,4]. The APE-related mortality rate is 7–11% within 30 days of diagnosis and rises to 30% in untreated cases [4]. These data show how important it is to perform an early diagnosis with easy-to-use, safe, and highly accurate diagnostic tools. The first hours after clinically suspected onset are critical [5].

Multidetector computed tomography pulmonary angiography (CTPA) is considered the method of choice for the imaging of pulmonary vascularisation. However, the low patient compliance, especially in cases of polytrauma, immobilisation, and assisted ventilation, as well as frequent cases of a presumed or ascertained allergic condition to the contrast medium, chronic renal failure, and concerns regarding pregnancy or fertility in women reduce its feasibility, especially in an emergency setting [6]. In addition, in some cases, CTPA is not conclusive with respect to the diagnosis of APE; further investigation is then required, often leading to inappropriate hospitalisation as a precautionary approach [7].

Lung perfusion scintigraphy (LPS) is a simple, easy-to-perform, and inexpensive nuclear medicine method with no contraindications or side effects, showing high performance for the diagnosis of APE in patients with high clinical suspicion but with inconclusive CTPA imaging. Due to its characteristics, LPS can be performed in all patients, including uncooperative and critical patients, as well as those with iodinated contrast medium (ICM) allergy and chronic renal failure. Furthermore, considering its very low irradiation, it can also be performed in young fertile or pregnant women [8].

### Importance and Objectives

The features reported above highlight the importance of LPS as a support in the diagnostic process of patients with suspected APE, providing elements that help to more clearly define the presence and the level of severity of embolism and, consequently, allowing the activation of the most appropriate therapeutic pathway.

Currently, the use of LPS in an emergency regimen is not carried out in all hospital centres. Moreover, to the best of our knowledge, no study has analysed the changes in healthcare pathways and the resources needed for the adoption of LPS to overcome the limits of other imaging methods in the healthcare decision-making process.

The aim of this study is thus to evaluate the economic and healthcare impact of adding LPS into the diagnostic pathway of suspected APE in the case of a non-conclusive or contraindicated CTPA examination, demonstrating, through our experience, the importance of a nuclear medicine service.

## 2. Materials and Methods

The current study was performed according to the Consolidated Health Economic Evaluation Reporting Guidelines (CHEERS) [9].

### 2.1. Patient Population—Organisation

We retrospectively evaluated patients with suspected APE and inconclusive or contraindicated CTPA who were admitted to the Nuclear Medicine Department, sent from the Medical Clinic, Surgery, or Emergency Room Department, to perform LPS in ordinary (from 8:00 a.m. to 4:00 p.m.) or emergency (the remaining 16 h) regimens [10].

The suspicion of APE was based on the presence of clinical symptoms (in particular, chest pain, dyspnoea, and cough), associated with altered biohumoral values (D-dimer dosage > 500 ng/mL), and abnormalities on the chest X-ray [11].

### 2.2. Lung Perfusion Scintigraphy (LPS): Acquisition and Interpretation Criteria

Lung perfusion scans were acquired using the OPTIMA NM/CT 670 or OPTIMA NM/CT 640 gamma camera (GE Medical System, West Milwaukee, WI, USA) soon after the intravenous injection of 185–370 MBq metastable technetium 99-labelled macroaggregated albumin (^99m^Tc-MAA) particles.

According to the European Association of Nuclear Medicine (EANM) guidelines [8], LPS was acquired via the planar technique in the anterior, posterior, right and left posterior/anterior oblique, and latero-lateral projections, using the following acquisition parameters: 128 × 128 matrix and 500–700 K counts per projection.

If necessary, an additional acquisition of LPS was performed using the single photon emission tomography (SPET) or SPET/CT techniques to obtain tomographic images with additional morpho-functional details.

In order to interpret LPS, the two criteria suggested by the Prospective Investigative Study of Acute Pulmonary Embolism Diagnosis (PISAPED) were used: (1) the presence of single or multiple wedge-shaped perfusion defects; and (2) the size of pulmonary perfusion defects (in the segmental or subsegmental region) [12].

### 2.3. Risk Stratification and Homecare

The Pulmonary Embolism Severity Index (PESI) has been used as a validated risk stratification score in predicting 30-day mortality for patients with PE and in identifying low-risk patients who can be discharged early for home treatment [13].

Based on patient characteristics, including the predictors that comprise the original PESI and the simplified PESI (sPESI) [14], risk assessment was performed in order to assign patients to the most appropriate therapy:

- High risk, for those patients who present shock or hypotension. Due to the high rate of in-hospital mortality, especially in the first hours after hospitalisation, they must be addressed to thrombolytic therapy or, if contraindicated or not enough, to surgical embolectomy. These patients need hospitalisation with intensive care;
- Intermediate risk, for those patients in apparent hemodynamic stability on admission showing signs of right ventricular dysfunction and/or myocardial injury. The initial preferred anticoagulant therapy is unfractionated intravenous therapy. These patients need ordinary hospitalisation;
- Low risk, for those patients without any primary APE-related risk factors for whom early discharge can be planned with adequate outpatient care and anticoagulant therapy can be provided (subcutaneous low-molecular-weight heparin; new oral anticoagulants).

### 2.4. Clinical Decision Models

Two alternative clinical decision models for the diagnosis of APE were compared in the present study for patients with inconclusive or contraindicated CTPA: with LPS (LPS pathway) and with CTPA alone (conventional pathway). These healthcare processes were represented through the standard graphical Unified Modeling Language^TM^ (UML^®^). UML was chosen based on the result of a systematic review of graphical languages/notation adopted in the healthcare setting, because it allows inter-professional analyses and is also easy to understand by non-experts [15]. Activity diagrams presented in this work were realised using Visual Paradigm Community Edition software [16].

**Figure 1:**
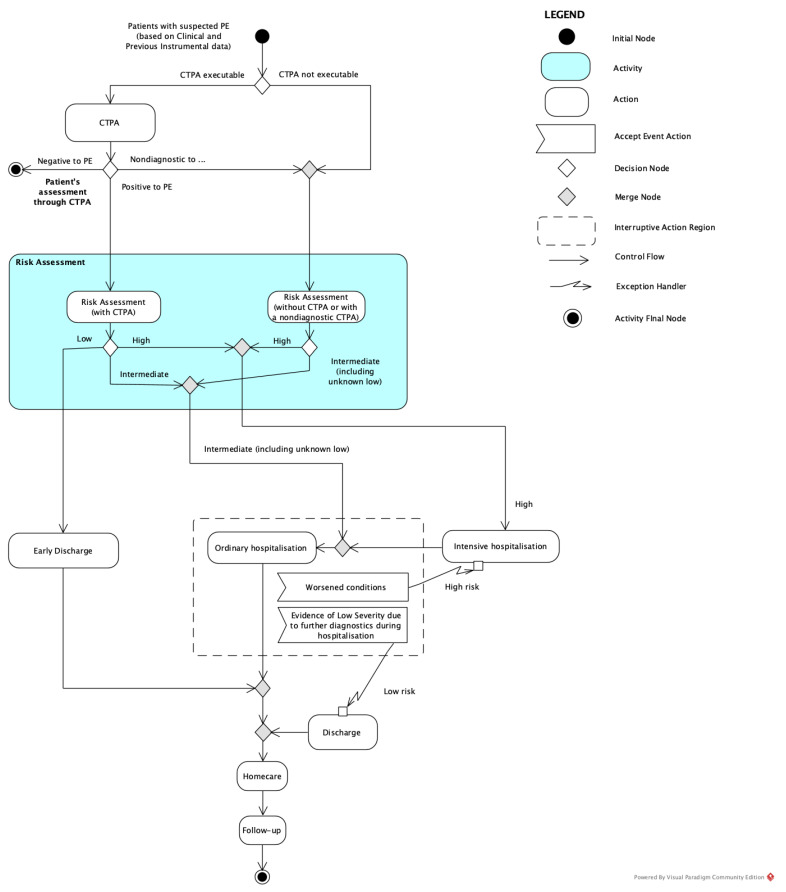
Representation of the conventional pathway: in the scenario where LPS is not performed, the patient is hospitalised both in the case of a CTPA-positive result and, precautionarily, in the case of non-conclusive or not-executable CTPA.

The conventional pathway requires that, in cases of suspected APE, the patient should undergo CTPA, if possible. If it is negative, and in the absence of significant clinical symptoms, the patient is sent home without further indications for APE unless other diseases are diagnosed. Conversely, the patient is hospitalised in the case of a positive result, as well as in the case of non-conclusive or not-executable CTPA (precautionarily) (see Figure 1).

**Figure 2:**
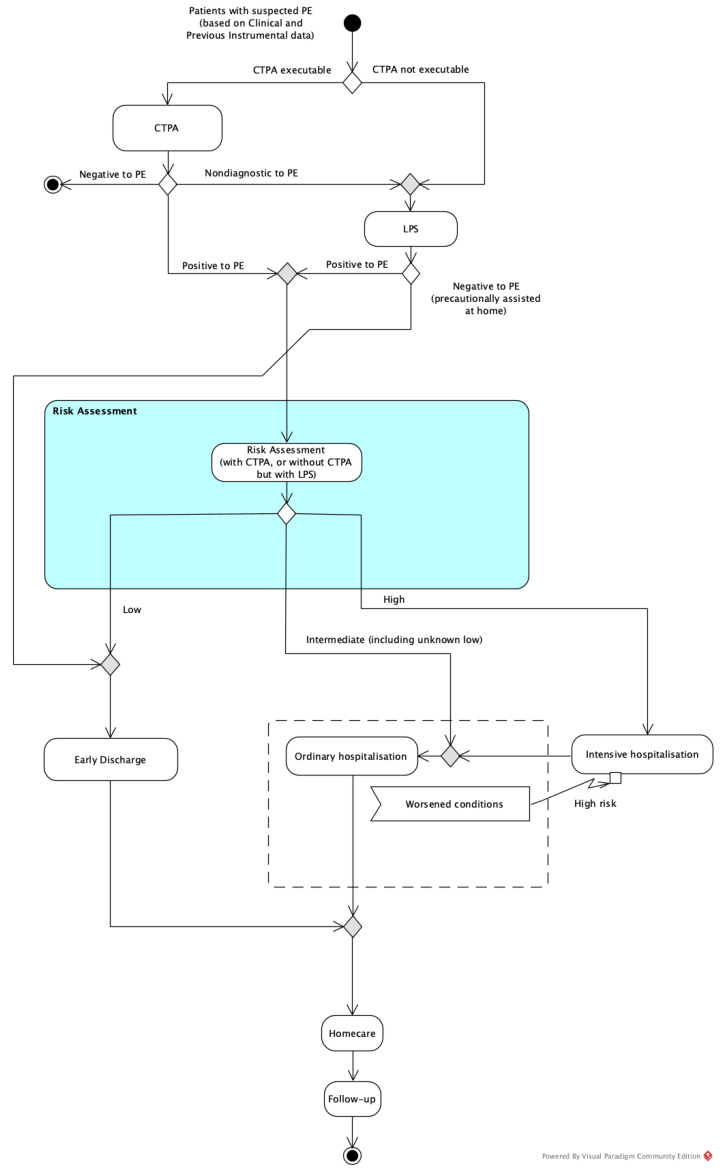
Representation of the LPS pathway: in the scenario where LPS is performed, in the case of non-conclusive or not-executable CTPA, the patient is precautionarily assisted at home if PE is negative, whereas they are admitted to the best risk-based hospitalisation regimen if PE is positive.

On the other hand, Figure 2 represents the process for patients with a non-conclusive or not-executable CTPA when LPS is available (LPS model).

Both clinical decision models include the possibility that (a) patients who undergo ordinary hospitalisation can incur a worsening of their condition and require a period in the Intensive Care Unit (ICU); and (b) every patient who undergoes intensive care requires subsequent ordinary hospitalisation before discharge.

### 2.5. Cost Analysis

Resource utilisation and costs were evaluated from the National Health Service (NHS) perspective and considered only direct healthcare costs.

The identification of costs was performed through retrospective data analysis considering the percentage of performed LPS, the percentage of LPS complemented by SPET/CT, and the average duration and daily cost for intensive care. In addition, we adopted official data that included national fees for ordinary hospitalisation for PE and diagnostic examinations. For the data relating to the percentage of subjects with an early discharge, we chose a value of 16.2%, in agreement with the literature [17].

The economic model was defined as a decision tree to evaluate the two described scenarios, conventional and LPS pathways, in the case of a non-executable or non-conclusive CTPA and considering hospital treatment of the acute phase after the risk assessment.

The outcome of the model was the per-patient cost for diagnosis, risk assessment, and treatment of the acute phase. To address significant uncertainty with the modelled parameters, sensitivity analyses were undertaken. Each parameter was varied through a range of plausible values, 25% either side of the base value.

## 3. Results

**Figure 3:**
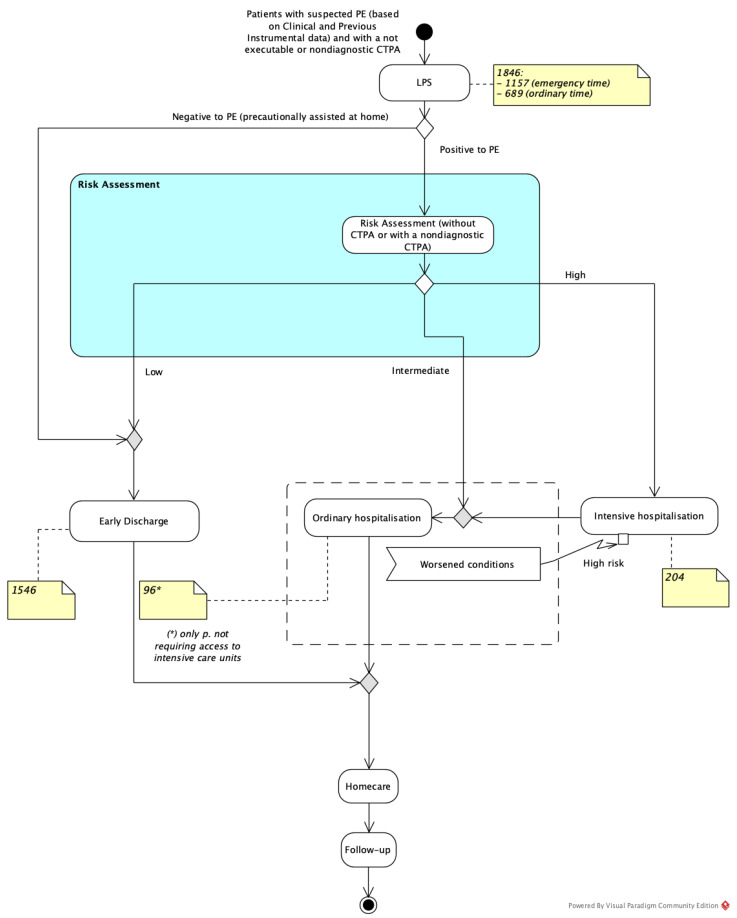
Patient flow within the diagnostic and therapeutic process for patients who underwent LPS, integrated with the results of our sample.

As reported in Figure 3, a total of 1846 instances of LPS were performed in our Nuclear Medicine Department over a period of four years, including patients from the Medical Clinic (*n* = 666), Surgery (*n* = 46), and Emergency (*n* = 1134) Departments. Of these, 1157 patients were admitted in our department during the emergency timeframe (from 4 p.m. to 8 a.m.), and the remaining 689 during the ordinary timeframe (from 8 a.m. to 4 p.m.). The majority of patients (1386/1846, 75%) could not undergo a CTPA examination because of allergic diathesis or immediate hypersensitivity reactions (IHRs) to iodinated contrast media (ICM), whereas 460/1846 (25%) patients underwent CTPA with a non-conclusive result. Planar LPS was integrated with SPET/CT in 369/1846 cases (20%). LPS results were positive in 309/1846 (16.7%) patients and negative in 1537/1846 (83.3%).

Out of the LPS-positive subjects, 9 were found to have a low severity and were sent home, while 300 were hospitalised. Among the patients requiring hospitalisation, 204/300 (68%) needed intensive care, whereas 96/300 (32%) required only ordinary hospitalisation (either in the Operational Unit of Cardiology or Pneumology Department).

The parameters considered in the model were derived from the cohort of our study and integrated with sources reported in Table 1.

Focusing on the scenario without LPS where PE low-risk patients with non-conclusive or not-executable CTPA are sent to ordinary hospitalisation precautionarily, our economic model assumed that the percentage of patients who were discharged after one day was 16.2% [17]. In fact, being discharged after one day was the critical parameter because it determined the application of a drastic cost reduction, moving from just a one-day ordinary hospitalisation cost of EUR 405 to an ordinary hospitalisation cost of EUR 4009 (see Table 1).

As detailed in Table S1a–c (in the Supplementary Material), the average per-patient costs for the diagnosis and treatment of acute phase PE in low-risk patients with a non-conclusive or not-executable CTPA, with and without LPS, are EUR 2145.25 and EUR 4912.45, respectively, with a difference of EUR 2767.20. Based on our study data, considering an annual patient flow of approximately 500 patients (1846 patients in four years) and applying a range of ±50% (from 250 to 750), the overall annual reduction in direct hospital costs could be estimated as varying from EUR 691,800 to EUR 2,075,400.

**Figure 4:**
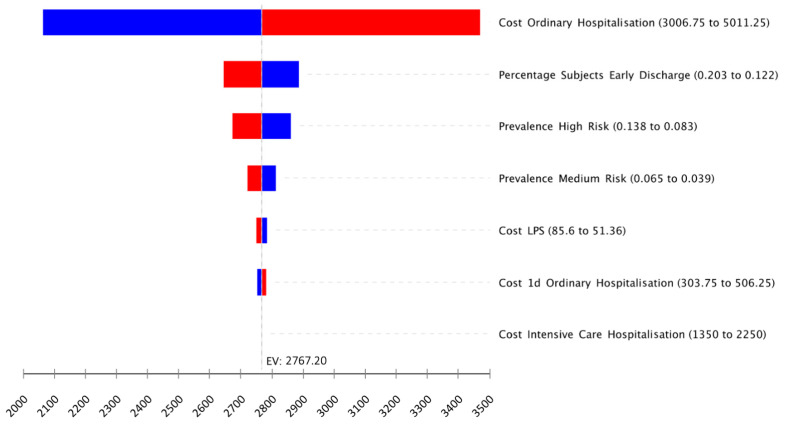
Tornado diagram of one-way sensitivity analyses of the differences between the two strategies. EV, expected value with baseline parameters; LPS, lung pulmonary scintigraphy. Blue bars indicate the effect of the parameter decrease.

Based on the sensitivity analysis, the most important driver of the cost difference between the two strategies is the cost of ordinary hospitalisation (Table 1a). When such cost is supposed to be low, at EUR 3006.75 (from EUR 4009.00 of the basal value), the overall expected per-patient cost difference decreases to EUR 2063.81 (EUR 1982.37 vs. EUR 4046.18 for the strategies with and without LPS, respectively). The findings were also sensitive to the percentage of subjects in ordinary hospitalisation who were discharged early. At its upper limit of 20.3%, the overall expected per-patient cost difference decreased to EUR 2643.45 (EUR 2145.25 vs. EUR 4788.70). However, the strategy with LPS remained superior in terms of cost savings compared with the alternative scenario for all the variables tested in the one-way sensitivity analysis (see Figure 4 for the six most important model variables).

**Figure 5:**
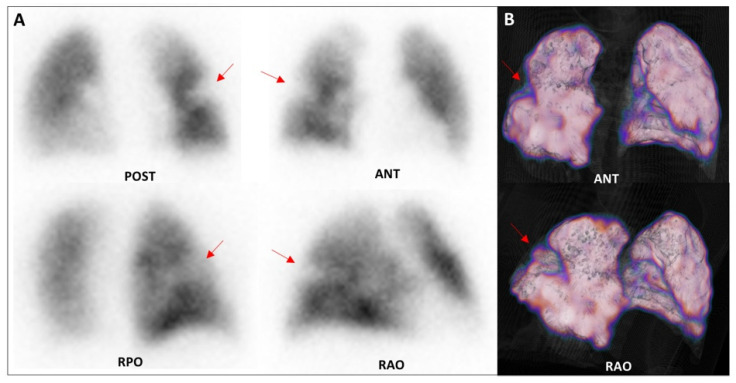
Lung perfusion scintigraphy performed for the suspicion of APE in a 79-year-old patient with dyspnoea, left chest pain, increased d-dimer values (842 ug/L, n.v. < 500), and chronic renal failure. Planar acquisitions (**A**) and tomographic 3D reconstruction (**B**) demonstrated a single wedge-shaped subsegmental perfusion defect in the posterior segment of the right upper lobe, thus confirming the suspicion of APE. Consequently, anticoagulant therapy was planned, and after one day of critical parameter monitoring, the patient was discharged early with adequate home therapy. POST: posterior; ANT: anterior; RPO: right posterior oblique; RAO: right anterior oblique.

**Figure 6:**
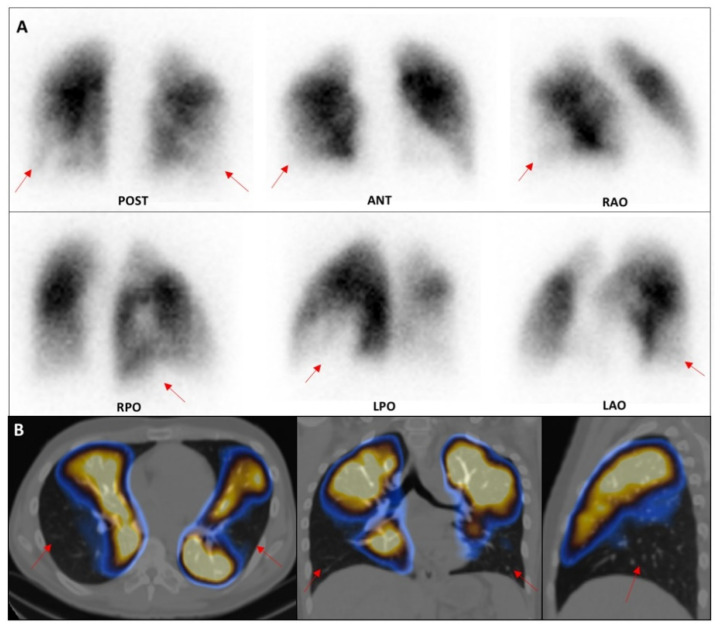
Lung perfusion scintigraphy performed for the suspicion of APE in a 32-year-old patient affected by a genetic rare disease (MTHFR mutation), who presented severe dyspnoea, hypoxia/hypocapnia at arterial blood gas analysis, increased d-dimer values (3253 ug/L, n.v. < 500), and referred allergic diathesis. Planar (**A**) and SPECT/CT (**B**) acquisitions demonstrated multiple wide wedge-shaped segmental perfusion defects in both lower lobes, thus confirming the suspicion of APE. Consequently, the patient was immediately referred to the intensive care unit for prompt therapeutic support. POST: posterior; ANT: anterior; RPO: right posterior oblique; RAO: right anterior oblique; LPO: left posterior oblique; LAO: left anterior oblique.

An example of the healthcare impact in terms of LPS detection ability is reported in the next two cases extracted from our sample of patients (Figure 5 and Figure 6). In both cases, LPS enabled the direct visualisation of lung perfusion through the identification of perfusion defects at the segmental/subsegmental level, providing information on the presence, site, and extent of embolism. The availability of modern SPET/CT technology offers the possibility of adding tomographic acquisition to the standard planar views, achieving higher sensitivity and specificity for a reliable diagnosis of APE in selected, more complicated, cases [16].

## 4. Discussion

APE is one of the most common and life-threatening cardiovascular conditions, with high morbidity and mortality [20]. Currently, multidetector CTPA is the examination of choice in patients with suspected APE, thanks to its high spatial resolution that provides detailed and optimal-quality imaging for studying the pulmonary vasculature, while also allowing a panoramic view of the whole chest and visualisation of possible different/concomitant diseases (such as atelectasis, bronchopulmonary foci, haemorrhagic foci, and/or emphysema), important for differential diagnosis [21].

However, the emergency APE scenario could reduce CTPA feasibility because it requires contrast medium administration, which is not always possible in patients with renal failure and/or allergic diathesis.

LPS is a nuclear medicine diagnostic tool with a consolidated role in supporting the diagnostic process of patients with suspected APE, thanks to its high performance, lack of contraindications or side effects, and very low irradiation exposure.

In fact, LPS provides an effective dose of 2.4 mSv for a 200 MBq administered dose, much lower than that provided by CTPA (14.4 mSv for a CTPA 16 Slice), leading to subsequent lower radiation doses to the lung and breast [22]. For this reason, LPS is considered the method of choice in young women of childbearing age and in pregnancy. In addition, it is increasingly used in the non-invasive assessment of APE in patients with renal insufficiency and contrast-medium allergy who cannot undergo CTPA, particularly in an emergency condition [6].

To the best of our knowledge, this study is the first to analyse the economic and healthcare impact of the use of LPS in the diagnostic pathway of suspected APE. All other studies have been more focused on evaluating the impact of target-specific oral anticoagulants [23] than on evaluating different potential pathways linked to alternative diagnostic tools.

As reported in Figure 1, our study assumed that, in a hospital without a nuclear medicine centre, all patients, whether unable to undergo a CTPA or with an indeterminate or uncertain diagnostic result, are hospitalised as a precaution and are kept under observation, with monitoring of the clinical course and symptoms, as well as laboratory tests, in order to confirm suspicions or to exclude pulmonary embolic pathology. In this way, although the patient is safe and constantly monitored by highly qualified medical personnel, there is the risk of an inappropriate hospitalisation, diagnosing the patient as a false positive, and then causing the administration of an unnecessary therapy regimen, with a consequent strong impact on national health expenditure.

The most relevant data emerging from the present study are that the more accurate patient stratification provided by LPS (among all 1846 patients included, with inconclusive or non-executable CTPA, 1537—83.3%—were negative for APE) determines an overall annual reduction in hospital direct costs estimated between EUR 691,800 and EUR 2,075,400 (EUR 2145.25 versus EUR 4912.45, respectively, for the strategies with and without LPS on a per-patient basis) thanks to early discharge and home treatment.

It appears clear that even in the less critical situation for the scenario without LPS, i.e., when 20.3% of inappropriate hospitalisation is resolved within just one day, the strategy with LPS is preferable in terms of costs: EUR 2145.25 (LPS pathway) vs. EUR 4788.70 (conventional pathway). If we consider a further increased economic burden for the LPS pathway, i.e., the need for SPET/CT integration, the average cost for a patient is minimally affected, as demonstrated in the sensitivity analysis (in fact, it is not among the first six relevant drivers of the cost difference between the two strategies). In addition to its low overall economic impact, SPECT/CT multimodality imaging provides additional morphological information, allowing a more accurate diagnosis to be obtained in a single-shot examination. With a significant part of healthcare costs linked to hospitalisations, our results are perfectly in line with the results reported in the literature [24].

In fact, the most recent European Society of Cardiology (ESC) guidelines, published in 2019, focus on the clinical management of PE. In particular, in the paragraph ‘Treatment in the acute phase’, a new recommendation of Class IIa (Level of evidence A) was inserted, which states that ‘carefully selected patients with low-risk PE should be considered for early discharge and continuation of treatment at home, if proper outpatient care and anticoagulant treatment can be provided’ [6]. Two meta-analyses confirmed the safety of home treatment in selected PE patients. Zondag et al. included 1657 PE patients who were treated at home as outpatients, finding low pooled incidences of recurrent VTE (1.7%, 95% CI 0.92–3.1), major bleeding (0.97%, 95% CI 0.58–1.59), and mortality (1.9%, 95% CI 0.79–4.8) that did not differ relevantly from those rates in hospitalised patients [25]. Piran et al. included 1258 patients and found these pooled incidences to be 1.47% (95% CI 0.47–3), 0.81% (95% CI 0.37–1.42), and 1.58 (95% CI 0.71–2.8), respectively [26]. These two meta-analyses demonstrated the safety of outpatient treatment and early discharge for patients judged as being at low risk of an adverse clinical outcome. Consequently, since 2014, international guidelines have indicated that early discharge and home treatment for selected acute low-risk PE patients with adequate home circumstances should be considered (Class IIa, Level of evidence B) [27]. Furthermore, in terms of healthcare impact, avoiding unnecessary hospitalisation is of utmost importance, especially in times of emergency that cause a shortage of beds reserved for typically managed diseases. The current very high demand for beds caused by the global novel coronavirus (SARS-CoV-2) pandemic makes any consideration on the re-determination of hospitalisation criteria useful.

Our data suggest that LPS, thanks to its high diagnostic accuracy in appropriately selecting patients, would enable us to avoid improper hospitalisations with huge economic savings, highlighting the great impact of 24 h nuclear medicine service availability. In addition, the sensitivity analysis could provide elements for reflection in scenarios with different numbers of patients assisted, epidemiological profiles, and costs of healthcare services.

This study also has some limitations. Firstly, it was a retrospective, single-centre study, and as such, our findings may be subject to selection bias or incomplete information. However, it is important to consider that our sample was derived from a very large hospital centre that covers a wide catchment area and a heterogenous population. To mitigate this limitation, we performed a sensitivity analysis on critical parameters to stress our economic model. Another limitation was the lack of follow-up data to check the current performance of LPS in the investigated cohort, with particular reference to false negative results. Nevertheless, the very high performance of LPS is well known. As described in the EANM guidelines, this examination has a negative predictive value (NPV) of 97–99%, a sensitivity of 96–99%, and a specificity of 96–98% for PE diagnosis, with a rate of nondiagnostic findings of just 1–4% [8], which is further decreased by the use of SPECT/CT, as in the modelled scenario. Among the most relevant studies in this setting, Sostman et al. reported an LPS specificity of 96.6% and NPV of 95.5% for acute APE, using PISAPED criteria [28]. These data were also confirmed by Mazurek et al., who reported significantly higher diagnostic accuracy with sensitivity, specificity, and NPV of 100%, 83%, and 100%, respectively, with the addition of the hybrid SPECT/CT technique [29]. For these reasons, even considering the low rate of false negative LPS results eventually included in our sample, our choice to consider early discharge and home treatment for selected acute low-risk PE patients is supported by the international guidelines [27].

## 5. Conclusions

LPS, when used to exclude the suspicion of APE in an emergency scenario, offers a huge advantage in terms of cost in health resources because it allows patients to avoid hospitalisation in favour of specialist outpatient follow-up, with a net benefit in terms of health resources in the territorial management of a critical pathology. Furthermore, this diagnostic tool proved to be of fundamental importance for better patient risk stratification and for optimisation of the management of those who do not require admission to intensive care or ordinary hospitalisation, with significant savings in terms of costs.

## Figures and Tables

**Table 1:** Parameters adopted in the economic model.

Name	Description	Basal Value	Sensitivity Range Values (±25%)	Source
Prevalence high risk	Prevalence of PE subjects needing hospitalisation in ICU	204/1846 (11.1%)	8.3%, 13.8%	Internal retrospective data
Prevalence medium risk	Prevalence of PE subjects needing ordinary hospitalisation	96/1846 (5.2%)	3.9%, 6.5%	Internal retrospective data
Percentage of subjects with early discharge	Percentage of subjects in ordinary hospitalisation who are discharged early, which determines a low fare	16.2%	12.2%, 20.3%	Stein et al. [17]
Daily cost of intensive care hospitalisation	Daily cost of hospitalisation for pulmonary embolism for patients requiring intensive care	EUR 1800	EUR 1350.00, EUR 2250.00	Expert opinion
Duration of intensive care hospitalisation	Number of days of hospitalisation for pulmonary embolism for patients requiring intensive care (average number)	7 days	/	Expert opinion
Cost of 1-day ordinary hospitalisation	Cost of the DRG 78 (pulmonary embolism) for patients discharged after 1 day	EUR 405	303.75 €, 506.25 €	Italian National Fares [18]
Cost of ordinary hospitalisation	Cost of the DRG 78 (pulmonary embolism) in ordinary hospitalisation	EUR 4009	EUR 3006.75, EUR 5011.25	Italian National Fares [18]
Cost of lung perfusion scintigraphy	Cost for executing an ordinary scintigraphy (i.e., during the morning regimen)	EUR 68.48	EUR 51.36, EUR 85.60	Italian National Fares [19]
Additional cost of pulmonary scintigraphy in emergency	Additional cost for executing an ordinary scintigraphy (i.e., during the evening/night regimen)	EUR 41.32	EUR 30.99, EUR 51.65	Internal retrospective data
Cost of SPET/CT supplemented LPS	The additional cost of an LPS when executed with SPET or SPET/CT	EUR 34.71	EUR 26.03, EUR 43.39	Italian National Fares [19]
Percentage of subjects with nondiagnostic LPS	Percentage of subjects with a nondiagnostic LPS that requires LPS complemented with SPET or SPET/CT	20%	15.0%, 25.0%	Internal retrospective data
Percentage of LPS in emergency	Percentage of LPS instances executed during the emergency timeframe	1157/1846 (62.7%)	47.0%, 78.3%	Internal retrospective data

## Supplementary Materials

The following are available online at https://www.mdpi.com/article/10.3390/healthcare9060716/s1, Table S1: One-way sensitivity analysis of the per-patient cost difference between the two strategies (top influencing variables); Table S2: One-way sensitivity analysis of the per-patient cost of the LPS strategy (top influencing variables); Table S3: One-way sensitivity analysis of the per-patient cost of the strategy not adopting LPS (top influencing variables).
