# Supplementary material for: Healthcare and Economic Impact of Lung Perfusion Scintigraphy in Patients Affected by Acute Pulmonary Embolism
Source: Healthcare (Basel). 2021 Jun 10;9(6):716. doi: 10.3390/healthcare9060716 (PMC8230672; doi:10.3390/healthcare9060716)
Supplement: Supplementary file 1 [file healthcare-09-00716-s001.zip › healthcare-1219416-supplementary.pdf]

healthcare

MDPI

Supplementary Material

Table S1. One-way sensitivity analysis of the per-patient cost difference between the two strategies (top influencing variables).

Model Variable					Impact on the measured value	Measured Value (basal case = 2,767.20€)		
Rank	Name	*Lowest* Value (-25%)	*Basal* Value	*Highest* Value (+25%)		With model variable at *Lowest* value	With model variable at *Highest* value	Variable Impact
1	Cost of ordinary hospitalisation	3,006.75 €	4,009.00 €	5,011.25 €	Increase	2,063.81 €	3,470.59 €	95.0%
2	Percentage subjects discharged after 1 day	12.2%	16.2%	20.3%	Decrease	2,887.93 €	2,643.45 €	2.9%
3	Prevalence of high-risk patients	8.3%	11.1%	13.8%	Decrease	2,861.42 €	2,673.04 €	1.7%
4	Prevalence of medium-risk patients	3.9%	5.2%	6.5%	Decrease	2,811.74 €	2,722.69 €	0.4%
5	Cost of LPS	51.36 €	68.48 €	85.60 €	Decrease	2,784.32 €	2,750.08 €	0.1%
6	Cost of 1d-lasting ordinary hospitalisation	303.75 €	405.00 €	506.25 €	Increase	2,753.46 €	2,780.94 €	0.0%

Table S2. One-way sensitivity analysis of the per-patient cost of the LPS strategy (top influencing variables).

Model Variable					Impact on the measured value	Measured Value (basal case = 2,145.25€)		
Rank	Name	*Lowest* value (-25%)	*Basal* Value	*Highest* Value (+25%)		With model variable at *Lowest* value	With model variable at *Highest* value	Variable Impact
1	Prevalence of high-risk patients	8.3%	11.1%	13.8%	Increase	1,688.35 €	2,601.85 €	58.1%
2	Cost of intensive care hospitalisation	1,350.00 €	1,800.00 €	2,250.00 €	Increase	1,797.15 €	2,493.36 €	33.7%
3	Cost of ordinary hospitalisation	3,006.75 €	4,009.00 €	5,011.25 €	Increase	1,982.37 €	2,308.13 €	7.4%
4	Prevalence of medium-Risk patients	3.9%	5.2%	6.5%	Increase	2,093.12 €	2,197.35 €	0.8%
5	Cost of LPS	51.36 €	68.48 €	85.60 €	Increase	2,128.13 €	2,162.37 €	0.1%

Table S3. One-way sensitivity analysis of the per-patient cost of the strategy not adopting LPS (top influencing variables).

Model Variable					Impact on the measured value	Measured Value (basal case = 4,912.45€)		
**Rank**	**Name**	***Lowest* value (-25%)**	***Basal* Value**	***Highest* Value (+25%)**		**With model variable at *Lowest* value**	**With model variable at *Highest* value**	**Variable Impact**
1	Cost of ordinary hospitalisation	3,006.75 €	4,009.00 €	5,011.25 €	Increase	4,046.18 €	5,778.72 €	73,7%
2	Prevalence of high-risk patients	8.3%	11.1%	13.8%	Increase	4,549.77 €	5,274.89 €	12,9%
3	Cost of intensive care hospitalisation	1,350.00 €	1,800.00 €	2,250.00 €	Increase	4,564.35 €	5,260.56 €	11,9%
4	Percentage subjects discharged after 1 day	12.2%	16.2%	20.3%	Decrease	5,033.18 €	4.788,70 €	1,5%
5	Cost of 1d-lasting ordinary hospitalisation	303.75 €	405.00 €	506.25 €	Increase	4,898.71 €	4,926.19 €	0.0%
